# Efficiency of 2017 ACR-TIRADS combined with contrast-enhanced ultrasound in diagnosing thyroid malignant nodules

**DOI:** 10.3389/fendo.2026.1776284

**Published:** 2026-07-28

**Authors:** Zhiqun Bai, Xi Chen, Yi Fang, Xuemei Wang, Zhen Zhang, Zhiguang Chen

**Affiliations:** 1 Department of Ultrasonic Diagnosis, the First Affiliated Hospital of China Medical University, Shenyang, Liaoning, China; 2 Department of Radiology, The Third Affiliated Hospital of Sun Yat-sen University, Guangzhou, Guangdong, China

**Keywords:** 2017 ACR-TIRADS, contrast-enhanced ultrasound, diagnostic accuracy, thyroid cancer, thyroid nodules, ultrasound

## Abstract

**Objective:**

The aim of this study was to compare the efficiency of 2017 ACR-TIRADS and contrast-enhanced ultrasound (CEUS) in diagnosing malignant thyroid nodules, and propose a new classification system based on CEUS and 2017 ACR-TIRADS.

**Methods:**

A total of 788 thyroid nodules, which were examined by conventional ultrasound and CEUS, were analyzed. The efficacy of contrast parameters in the diagnosis of benign and malignant thyroid nodules was analyzed by univariate analysis and logistic regression. The contrast parameters were assigned based on the results of logistic regression analysis, and subsequently, a new classification system was proposed.

**Results:**

1) The result of univariate analysis showed that non-uniform enhancement, hyper-enhancement, fast entry, and washout were significant differences between benign and malignant nodules. 2) When taking ≥ 7 points as the cutoff value for diagnosing malignant thyroid nodules, the sensitivity and specificity of the 2017 ACR-TIRADS were 87.27% and 55.06%, respectively; whereas the sensitivity and specificity of the combined CEUS and ACR-TIRADS were 86.41% and 57.30%, respectively; 3) By incorporating the 2 points derived from CEUS into the 2017 ACR-TIRADS assignment process, a new grading system, namely CEUS-TIRADS, was established. The sensitivity and specificity of CEUS-TIRADS were 87.27% and 57.30% respectively.

**Conclusion:**

The combined application of CEUS and 2017 ACR-TIRADS demonstrated better diagnostic efficiency than their individual application. Incremental improvement in diagnostic specificity and Youden index compared with their individual application, although the difference did not reach statistical significance. The CEUS-TIRADS offers a preliminary scoring approach and a new research direction for future prospective studies to analyze quantitative and qualitative CEUS data and establish a validated CEUS classification system for thyroid nodules.

**Advances in knowledge:**

The combined application of CEUS and 2017 ACR-TIRADS provides a preliminary scoring framework and a promising research direction for the future development of a validated CEUS-based classification system for thyroid nodules, pending external validation in larger cohorts.

## Introduction

With the advancement of imaging technology, especially the application of high-resolution ultrasonography, an increasing number of thyroid nodules have been detected. The incidence rate of thyroid nodules reaches to 65% in general population (1). Although thyroid cancer is the most common endocrine carcinoma, with an incidence rate of approximately 2.1% of all cancers in the world, it only constitutes a small part of thyroid nodules (about 5%) (1, 2). Therefore, it is imperative to accurately screen malignant nodules accurately and intervene appropriately. Ultrasound is a non-invasive, real-time and portable imaging tool that is widely used in the detection, diagnosis and treatment of thyroid nodules (3). To date, several ultrasonic signs have been recognized to be related to malignant nodules and recommended by guidelines, including solid composition, hypoechoic or very hypoechoic appearance, a taller-than-wide shape, irregular edges, and microcalcifications (4–6). However, none of these is both sensitive and specific enough to exclude or confirm thyroid malignancy (7).

The proposal of the thyroid imaging reporting and data system (TI-RADS) provides Guidance on the management of thyroid nodules (8). In 2017, the American College of Radiology updated its 2015 guidelines to create the 2017 ACR-TIRADS (5). It has been widely applied in clinical practice and offers higher specificity, reducing the number of unnecessary biopsies for benign nodules compared to other risk stratification systems (9). Compared to Color-Doppler and superb microvascular imaging (SMI), contrast-enhanced ultrasound (CEUS) is more sensitive in displaying the blood perfusion pattern in the region of interest, particularly in revealing microvascularization and perfusion heterogeneity, which are critical for distinguishing between benign and malignant thyroid nodules (10–12). With the increasing detection of thyroid nodules, accurate differentiation between benign and malignant nodules is crucial. The 2017 ACR-TIRADS system has been widely used, but its diagnostic accuracy can be further improved. This study aims to evaluate the efficacy of combining CEUS with 2017 ACR-TIRADS in diagnosing malignant thyroid nodules and to propose a new classification system based on CEUS and ACR-TIRADS.

This clinical scenario presents a diagnostic paradox: thyroid nodules that most urgently require accurate risk stratification—those with indeterminate or borderline sonographic features (e.g., TIRADS 3 or 4)—are precisely those where conventional ACR-TIRADS has the greatest diagnostic uncertainty. In contrast, clearly benign nodules (TIRADS 1-2) and overtly malignant nodules (TIRADS 5) are easily classified without additional imaging. This paradox directly impacts patient selection for FNA biopsy: patients with indeterminate nodules often face unnecessary invasive procedures or diagnostic delays, while those with suspicious nodules may benefit from additional functional imaging information to guide management. CEUS, by providing real-time hemodynamic information about nodule microvascularization, has the potential to resolve this diagnostic uncertainty and improve the risk stratification of indeterminate thyroid nodules, thereby reducing unnecessary FNA procedures and optimizing clinical decision-making.

Previous studies have consistently demonstrated that CEUS features—particularly enhancement uniformity, enhancement intensity, and wash-in/wash-out patterns—differ significantly between benign and malignant thyroid nodules (10-12). Several investigators have also explored the combination of CEUS with various TIRADS systems and reported improved diagnostic performance (11, 12). However, a critical gap remains: there is currently no standardized, easily implementable scoring system that integrates CEUS parameters into TIRADS using an objective weighting method. Most existing approaches rely on subjective interpretation or complex machine-learning algorithms that are not readily applicable in routine clinical practice. Our study addresses this gap by proposing a novel odds ratio-based CEUS-TIRADS scoring system that simplifies CEUS parameter interpretation, enables reproducible risk stratification, and can be easily adopted by clinicians without specialized software.

The aim of this study was to evaluate the efficacy of CEUS and 2017 TI-RADS, both individually and in combination, in diagnosing thyroid malignant nodules, and to propose a new classification method based on CEUS and TI-RADS.

## Patients and methods

### Ethics approval and consent to participate

This retrospective study was approved by the Ethics Committee of the First Affiliated Hospital of China Medical University ([2025] 584). The study complied with the Declaration of Helsinki. All patients had provided oral or written informed consent for the biopsy procedure as part of the standard clinical protocol. The Ethics Committee granted a waiver of additional informed consent for this retrospective analysis of anonymized clinical data.

### Study group

A total of 788 nodules, which obtained definite pathological results and were examined by CEUS and conventional ultrasound, were retrospectively analyzed from January 2016 to January 2022 in the Department of Ultrasound. Among these nodules, 89 were benign and 699 were malignant. A total of 753 patients, aged 18-79 years (average age 43.71 ± 12.00 years), were included. There were 194 males and 559 females.

The inclusion criteria were as follows: (i) nodules confirmed by surgical histopathology (n=720) or by FNA with Bethesda category II or VI (n=68); (ii) patients who underwent surgical resection were definitively diagnosed as benign nodules or thyroid cancer postoperatively; and (iii) the interval between CEUS and surgery or fine-needle aspiration (FNA) did not exceed 2 weeks. The reference standard was surgical histopathology for 720 nodules and FNA cytopathology (Bethesda category II for benign, Bethesda VI for malignant) for 68 nodules. While Bethesda II nodules are generally considered benign with a very low residual malignancy risk (approximately 0-3%), we acknowledge this as a potential source of misclassification, which is further addressed in the Limitations section.

The exclusion criteria were as follows: (i) lack of CEUS; (ii) absence of pathological results; (iii) the Bethesda system for reporting thyroid cytopathology indicated Bethesda I, III, IV, or V; and (iv) CEUS showed that the nodules were not consistently enhanced.

**Figure 1:**
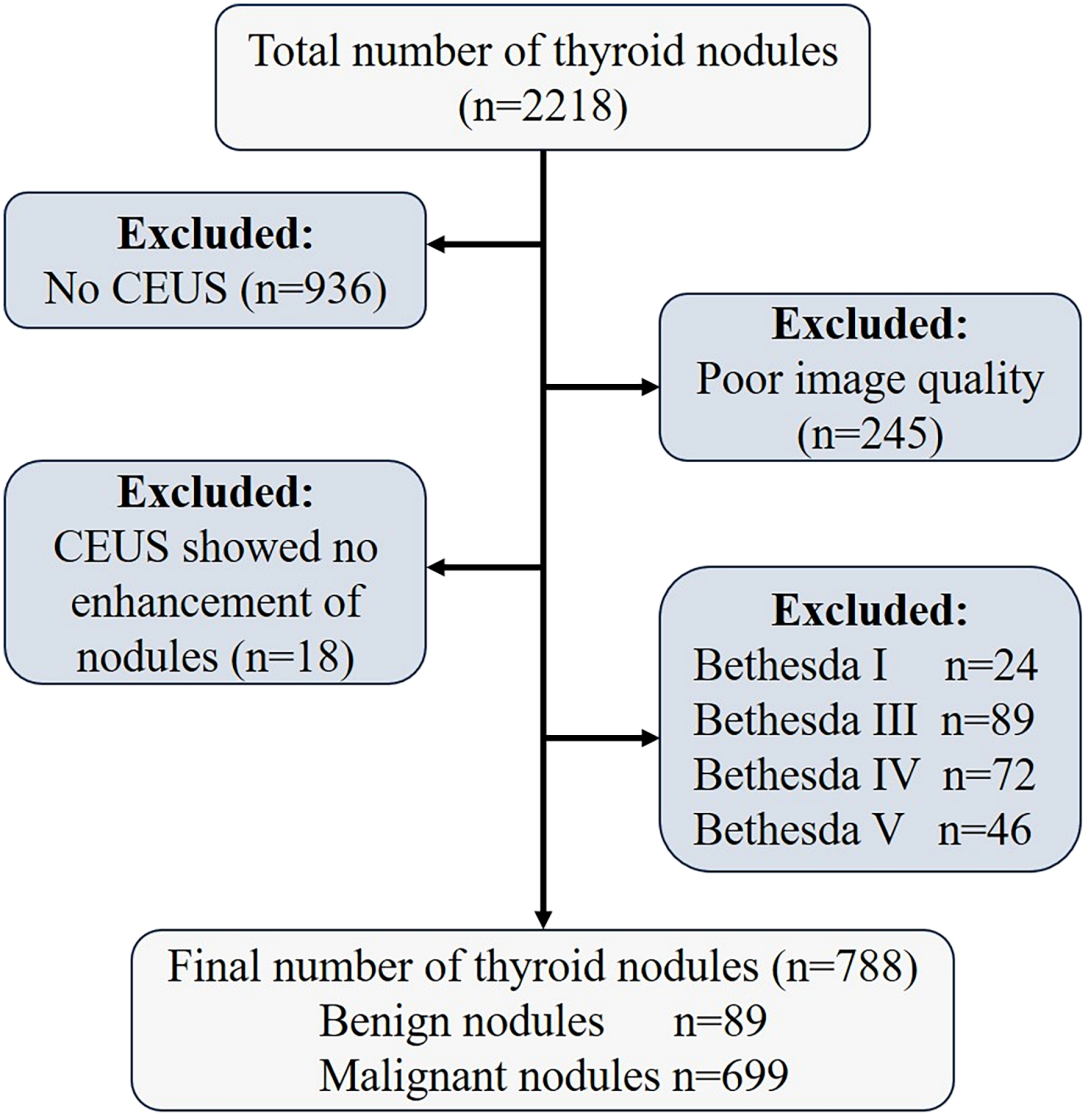
Flow chart of participant screening and nodule inclusion/exclusion.

The process of thyroid nodules selection is shown in **Figure 1**.

### Ultrasonography and CEUS

Preirus^®^ (linear array probe =5 – 12MHz; Hitachi, Tokyo, Japan), AixPlorer^®^ (linear array probe =4 – 15 MHz; Supersonic, France), Aplio i900^®^ (linear array probe = 18 MHz; Canon, Japan).

Firstly, all the patients underwent the conventional ultrasonography. According to the 2017 ACR-TIRADS (5), the following sonographic signs of thyroid nodules were recorded.

1. Composition, which including solid or almost completely solid, mixed cystic and solid, spongiform, and cystic or almost completely cystic.
2. Echogenicity which including anechoic, very hypoechoic, hypoechoic, isoechoic, and hyperechoic.
3. Shape which including taller-than-wide and wider than tall.
4. Margin which including smooth, Ill-defined, lobulated or irregular, and extra-thyroidal extension.
5. Echogenic foci which including microcalcifications, large comet-tail artifacts, macrocalcifications, and peripheral calcifications.

CEUS was performed under the contrast-specific imaging mode. The contrast agent used was SonoVue (Bracco S.p.A. Milan, Italy), containing 25 mg of lyophilized powder and 59 mg of sulfur hexafluoride (SF_6_) gas. The lyophilized powder was dissolved in 5 mL of 0.9% sodium chloride solution immediately before use. A bolus of 2.4 mL of the reconstituted suspension was injected through an antecubital vein, followed immediately by a 5 mL saline flush. A low mechanical index (< 0.12) was maintained throughout the examination to minimize microbubble destruction. Continuous cine clips were recorded from 10 seconds to 3 minutes after contrast injection for subsequent offline analysis.

**Figure 2:**
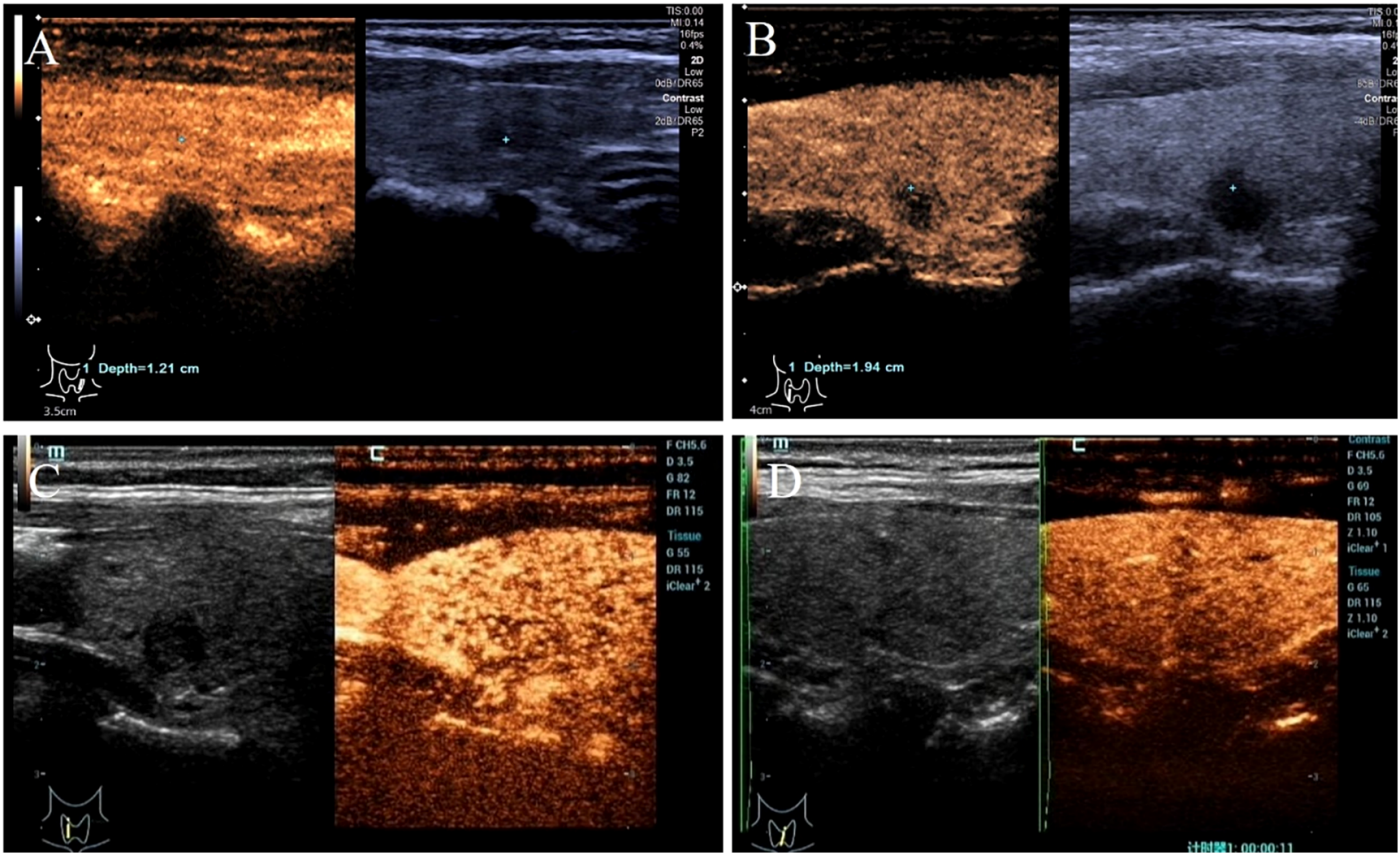
Representative CEUS images of thyroid nodules showing different enhancement patterns. **(A)** Uniform enhancement; **(B)** Non-uniform low enhancement; **(C)** Non-uniform high enhancement; **(D)** Uniform low enhancement. CEUS was performed using SonoVue contrast agent with a linear array probe. The enhancement patterns were independently evaluated by two experienced radiologists.

Four contrast parameters were evaluated for each nodule (**Figure 2**):

1. Enhanced phase — the timing of contrast arrival relative to the surrounding thyroid parenchyma, categorized as fast entry (arrival earlier than parenchyma), equal entry (arrival synchronous with parenchyma), or slow entry (arrival later than parenchyma).
2. Peak enhancement — the maximum enhancement intensity of the nodule relative to the surrounding parenchyma, categorized as hyper-enhancement (greater than parenchyma), iso-enhancement (similar to parenchyma), or hypo-enhancement (less than parenchyma).
3. Fading phase — the washout pattern after peak enhancement, categorized as rewind down(rapid washout), retreating (gradual washout), or slow back (slow washout).
4. Enhanced uniformity — the homogeneity of intranodular contrast distribution, categorized as uniformity (homogeneous enhancement throughout the nodule) or non-uniformity(heterogeneous enhancement with visible perfusion defects).

Two ultrasound physicians (BZQ and FY with 3 and 5 years of thyroid ultrasound experience, respectively) independently evaluated all CEUS images. In cases of disagreement (approximately 6.7% of nodules), a senior radiologist (Professor CZG, with 8 years of experience) reviewed the images and made the final decision. All interpreters were blinded to the pathological results and the ACR-TIRADS scores to avoid bias.

### The assignment process for the new classification system

Firstly, we performed univariate analysis on the contrast parameters of benign and malignant nodules, followed by logistic regression analysis on the significant univariate analysis results. We established a logistic regression model to calculate the number of benign and malignant nodules diagnosed by CEUS, and compared these results with the pathological gold standard to determine the odd ratio (OR) for malignant nodules. Then, we assigned the contrast parameters according to the OR assignment method. Two ultrasound doctors (BZQ and CX) independently evaluated the ultrasonic signs. In case of disagreement, senior doctors (Professor CZG) were consulted for a final decision. The ultrasound doctors interpreting the ultrasound studies were blinded to the pathology findings to ensure unbiased evaluation.

Secondly, the CEUS assignment parameters were incorporated into the 2017 ACR-TIRADS, which involved reassigning conventional ultrasound signs and CEUS parameter. The scores for each nodule were then calculated, and the diagnostic efficiency of the new classification system was assessed.

### The assignment based on OR

If OR < 0.3, there was no correlation with malignancy. If 0.3 <OR ≤ 3, there was a nonsignificant correlation with malignancy. If 3 <OR ≤ 10, there was a strong correlation with malignancy. If OR >10, there was an extremely strong correlation with malignancy. Based on these scenarios, the assignments were 0, 1, 2, or 3 points, respectively (13).

### Statistical analysis

Statistical analysis was conducted using SPSS v23 (IBM, Armonk, NY, USA). Measurement data were presented as mean ± SD, while categorical variables were presented as frequencies.

We performed univariate analysis of contrast parameters using the chi-square test for categorical data and the independent sample t-test for measurement data. The diagnostic consistency between BZQ and JZY was tested using the Intraclass Correlation Coefficient (ICC). The significant univariate analysis results were used for multivariate analysis to develop a model for differentiating malignant and benign thyroid nodules. The diagnostic efficiency of the 2017 ACR-TIRADS, CEUS alone and their combination was analyzed. A receiver operating characteristic (ROC) curve was then plotted to determine the optimal diagnostic cut-off point. A *P-value* < 0.05 was considered statistically significant.

## Results

### The results of CEUS in diagnosing thyroid malignant nodules

In malignant nodules, 91.70% (641/699) exhibited non-uniform enhancement, 74.96% (524/699) showed hyper-enhancement, 68.81% (481/699) demonstrated slow entry during the enhanced phase, and 69.10% (483/699) exhibited a rewind effect during the fading phase. In benign nodules, 74.16% (66/89) exhibited non-uniform enhancement, 60.67% (54/89) showed hyper-enhancement, 52.81% (47/89) demonstrated slow entry during the enhanced phase, and 53.93% (48/89) exhibited rewind effect during the fading phase. The inter-rater consistency between the two ultrasound physicians was good, with an ICC of 0.895. Significant differences in nodule contrast parameters were observed between the two groups (*P* < 0.05) (**Table 1**).

**Table 1:** The univariate analysis results of CEUS.

Groups	Enhanced uniformity		Peak enhancement				Enhanced phase			Fading phase		
	Non-uniformity	Uniformity	Hyper-	Iso-	Hypo-		Fast entry	Equal entry	Slow entry	Rewind down	Retreating	Slow back
M(n=699)	641	58	524	122		53	42	176	481	483	198	18
B (n =89)	66	23	54	24		11	14	28	47	48	36	5
χ^2^	26.351		8.272				14.721			9.107		
P	0.0001		0.016				0.001			0.011		

The contrast parameters presented in **Table 1** were included in the logistic regression analysis. According to the logistic regression results, non-uniform enhancement and fast entry emerged as independent predictors of malignant thyroid nodules. Thus, the logistic regression model was derived as follows: Y = 1.32 * X_1_ + (- 1.33) * X_2_ + 1.45, where X_1_ represents non-uniform enhancement and X_2_ represents fast entry. The values of X_1_ and X_2_ are binary (1 or 0), with 1 indicating the presence of non-uniform enhancement or fast entry, and 0 indicating uniform enhancement, equal entry, or slow entry (**Table 2**).

**Table 2:** Logistic regression results of CEUS.

Contrast parameters	Regression coefficient	Standard error	Wald value	*P*	OR	95%CI	
						Low	Up
Non-uniformity	1.32	0.35	14.06	**0.00**	3.76	1.88	7.51
Hyper-	-0.88	0.61	2.09	0.15	0.41	0.13	1.37
Iso-	-0.87	0.61	2.08	0.15	0.42	0.13	1.37
fast entry	-1.33	0.59	5.07	**0.02**	0.26	0.08	0.84
equal entry	0.15	0.46	0.10	0.75	1.16	0.47	2.87
rewind down	0.48	0.63	0.56	0.45	1.61	0.46	5.57
retreating	0.25	0.63	0.16	0.69	1.29	0.38	4.41
constant	1.45	0.88	2.73	0.10	4.28	-	–

Bold values of *P* denote statistically significant associations at the level of *P* < 0.05.

**Figure 3:**
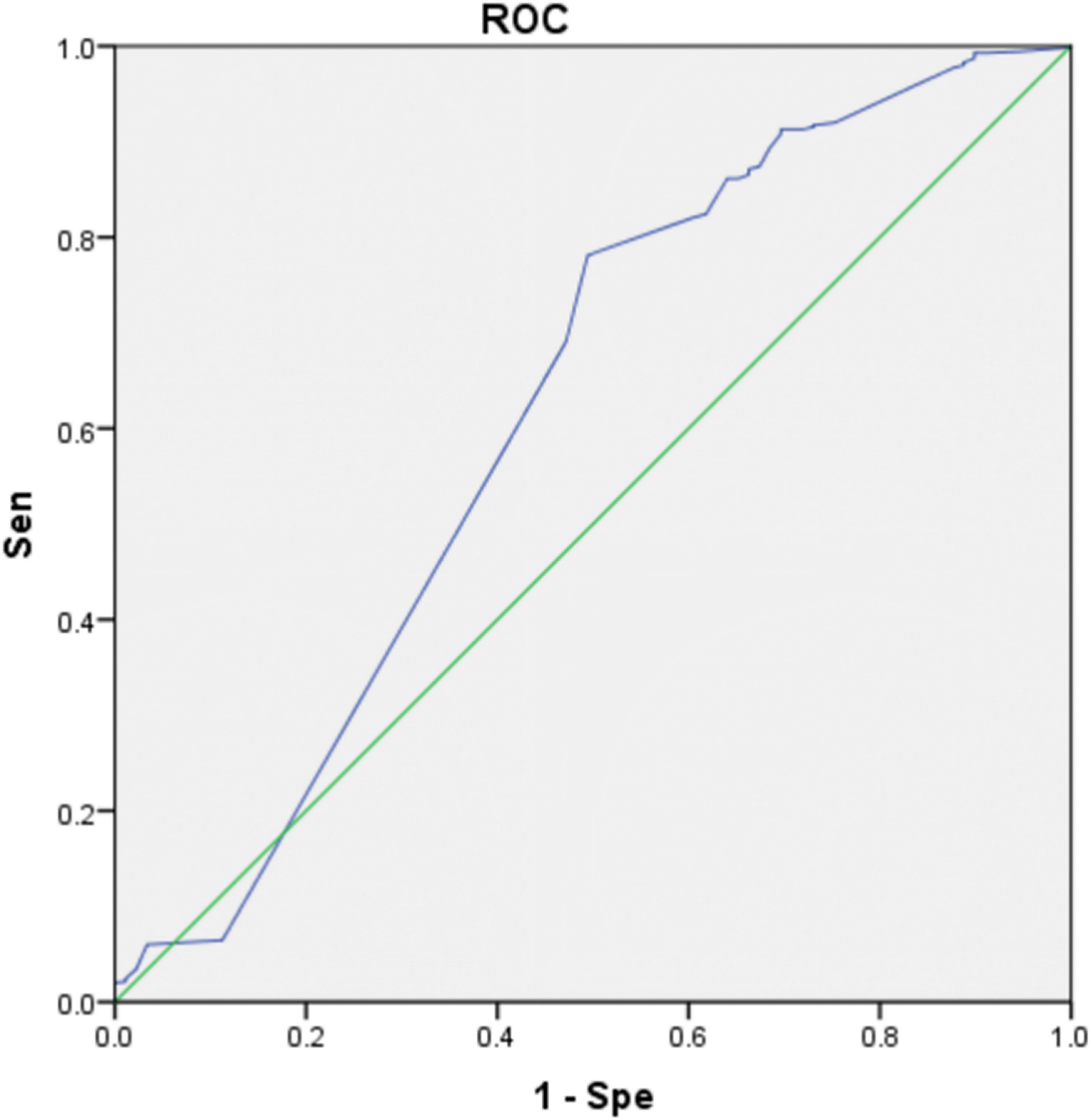
ROC curve for CEUS alone in differentiating benign from malignant thyroid nodules.

According to the logistic regression results, an ROC curve was established, and the area under the ROC curve (AUC) was 0.617; When Y ≥ 0.909 was taken as the cutoff value for the diagnosis of malignant thyroid nodules, the sensitivity and specificity of CEUS were 78.11% and 50.56%, respectively (**Figure 3**).

### The results of 2017 ACR-TIRADS in diagnosing malignant thyroid nodules

We classified 788 nodules according to 2017 ACR-TIRADS, 1 nodule was ACR-TIRADS 1, with a malignant rate of 0. There were 9 nodules were ACR-TIRADS 2, and the malignant rate was 11.11% (1/9). There were 10 nodules were ACR-TIRADS 3, and the malignant rate was 20.00% (2/10). There were 118 were ACR-TIRADS 4, and the malignant rate was 72.88% (86/118). There are 650 nodules were ACR-TIRADS 5, and the malignant rate was 93.85% (610/650), as shown in **Table 3**.

**Table 3:** The classified result of ACR-TIRADS.

Category	ACR-TR 1	ACR-TR 2	ACR-TR 3	ACR-TR 4	ACR-TR 5
Total nodules(n)	1	9	10	118	650
B/M(n)	1/0	8/1	8/2	32/86	40/610
Actual malignant rate(%)	0	5.99	20.00	72.88	93.85
Predicted malignancy rate(%)	< 2	< 2	< 5	5 - 20	≥ 20

B/M, the number of benign nodules/the number of malignant nodules.

**Figure 4:**
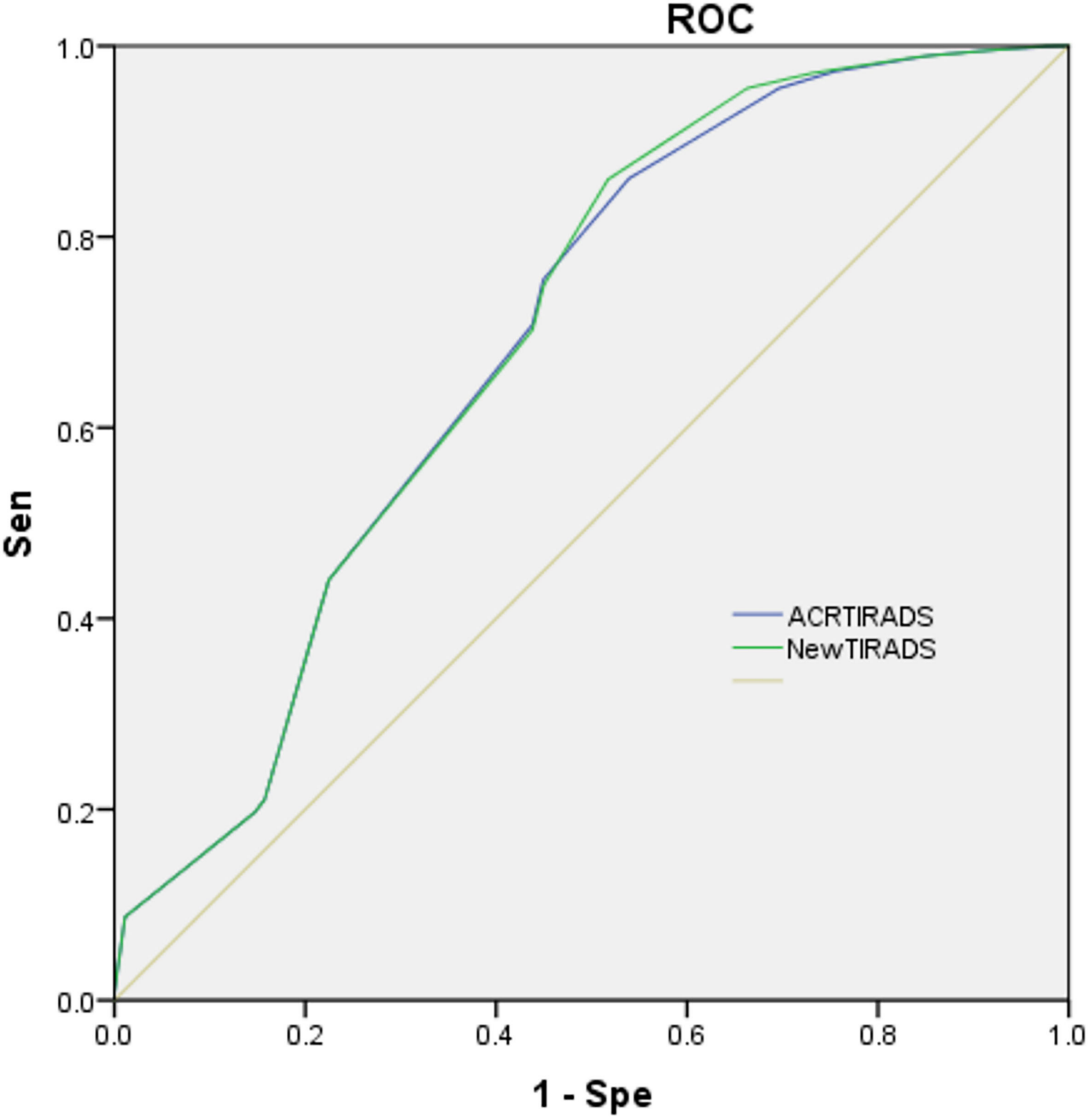
Comparative ROC curves of 2017 ACR-TIRADS vs CEUS-TIRADS.

The ROC curve was established based on the scoring results of 2017 ACR-TIRADS, and the AUC was 0.689. When taking ≥ 7 points as the cutoff value for the diagnosis of malignant thyroid nodules, the diagnostic sensitivity and specificity of ACR-TIRADS were 87.27% and 55.06%, respectively (**Figure 4**).

### The results of 2017 ACR-TIRADS combined with CEUS in diagnosing malignant thyroid nodules

In the logistic regression model, Y = 1.32 * X_1_ + (- 1.33) * X_2_ + 1.45; (X_1_ refers to the enhanced uniformity; X_2_ refers to the enhanced phase). By substituting X_1_ and X_2_ into the model, if Y ≥ 0.909, CEUS classified the nodule as malignant; otherwise, it was considered benign. CEUS results showed that 198 of 788 nodules may be benign and the remained 590 may be malignant.

According to the 2017 ACR-TIRADS, nodules with a score ≥ 7 was considered malignant, specifically those classified as ACR-TIRADS 5, totaling of 650 nodules. On the contrary, nodules classified as ACR-TIRADS 1, ACR-TIRADS 2, ACR-TIRADS 3, and ACR-TIRADS 4 were deemed benign, totaling 138 nodules.

Nodules diagnosed as malignant by both CEUS and the 2017 ACR-TIRADS were classified as jointly malignant, while those not diagnosed as malignant by both were considered benign. A total of 604 nodules were classified as malignant, and 184 as benign. The sensitivity and specificity of the combined diagnosis were 86.41% and 57.30% respectively (**Table 4**).

**Table 4:** Efficiency comparison of several diagnostic methods.

Parameters	CEUS	ACR-TIRADS	CEUS+ACR	CEUS-TIRADS
Sen (%)	78.11	87.27	86.41	87.27
Spe (%)	50.56	55.06	57.30	57.30
Accuracy (%)	75.00	83.63	83.12	83.33
PPV (%)	92.54	93.84	94.08	94.13
NPV (%)	22.73	35.51	34.93	36.63
Youden index	0.2867	0.4233	0.4371	0.4457

There was no statistically significant difference in diagnostic efficacy among the four diagnostic combinations (χ^2^ = 0.359, *P* = 0.949). CEUS-TIRADS demonstrated the highest Youden index (0.4457) among the four methods, indicating a slight incremental improvement in overall diagnostic performance (**Table 4**). The AUC for CEUS-TIRADS was 0.692, as shown in **Figure 4**.

### The results of the newly TI-RADS in diagnosing malignant thyroid nodules

Based on CEUS results, the OR for diagnosis malignant nodules was 5.884. Specifically, when the nodule exhibiting uniform enhancement, equal entry, or slow entry were considered benign. Whereas those not exhibiting these features were considered malignant. Subsequently, malignant nodules were assigned as 2 points (using the OR assignment method). On the basis of the assignment result of 2017 ACR-TIRADS, we added the score of CEUS to get a new TI-RADS, namely CEUS-TIRADS.

The ROC curve (**Figure 3**) was constructed based on CEUS-TIRADS. The AUC was 0.692, with a sensitivity of 87.27% and a specificity of 57.30% for diagnosing malignant thyroid nodules.

According to the assignment results, we calculated the score of each nodule and counted the malignant rate corresponding to each score. **Table 5** showed the comparison with CEUS-TIRADS assignment results and malignant rate.

**Table 5:** the malignant rate of each score in CEUS-TIRADS.

Score		2	4	5	6	7	8	9	10	11	12	13	14	15	16
CEUS	Benign	2	7	9	12	7	14	8	3	14	6	0	5	2	0
	malignant	0	1	3	10	10	66	76	30	188	161	11	83	54	6
Malignant rate(%)		0	12.50	25.00	45.45	58.82	82.50	90.48	90.91	93.07	96.41	100	94.32	96.43	100

**Figure 5:**
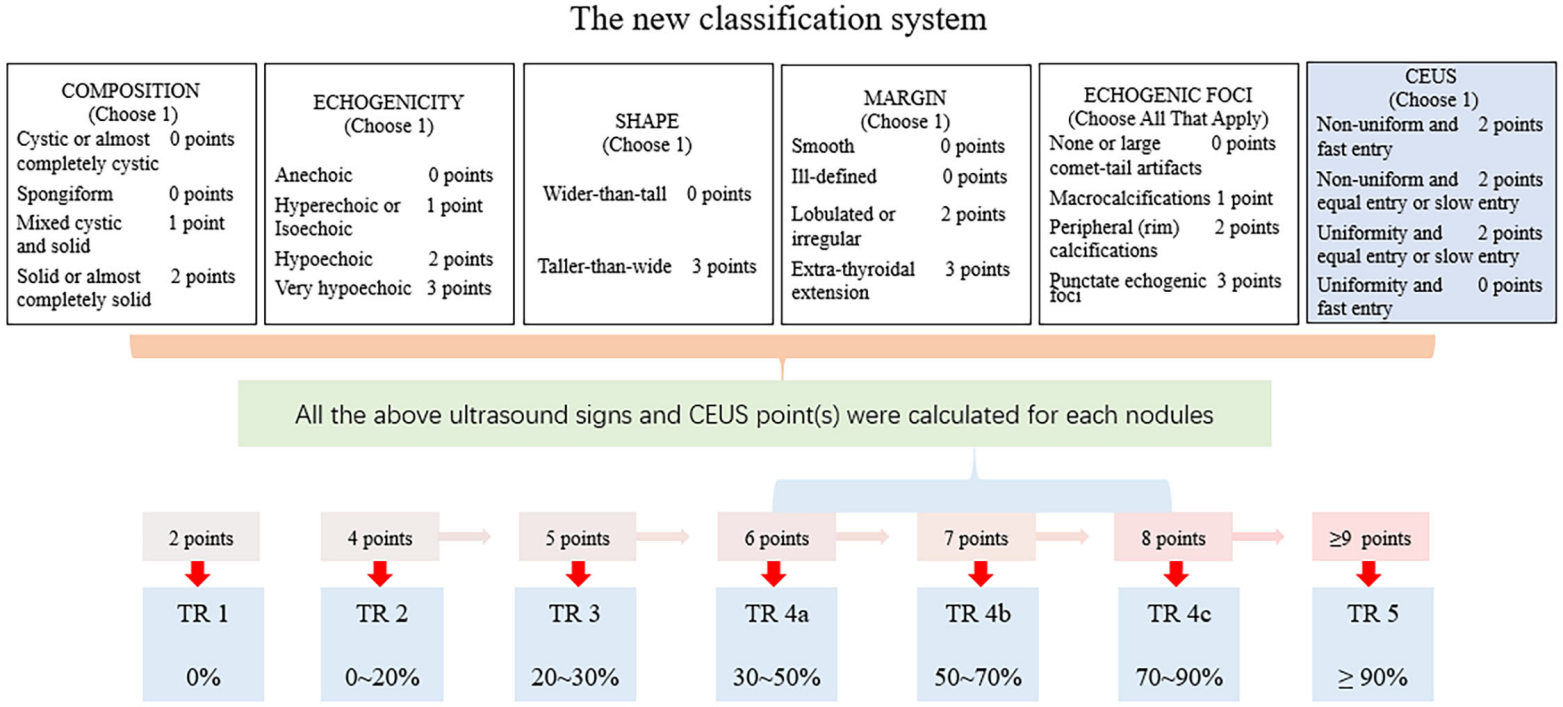
Scoring framework and risk stratification flowchart of the novel CEUS-TIRADS system.

Referring to the 2017 ACR-TIRADS classification method, we divided CEUS-TIRADS into CEUS-TIRADS 1, score 2 and malignant rate 0; CEUS-TIRADS 2, score 4 and malignant rate 0-20%; CEUS-TIRADS 3, score 5 and malignant rate 20% - 30%; CEUS-TIRADS 4a, score 6 and malignant rate 30% - 50%; CEUS-TIRADS 4b, score 7 and malignant rate 50% - 70%; CEUS-TIRADS 4c, score 8 and malignant rate 70% - 90%; CEUS-TIRADS 5, score ≥ 9 and malignant rate ≥ 90%, **Figure 5**.

## Discussion

Active surveillance may be an alternative management method for thyroid cancer, and existing guidelines do not recommend FNA for nodules ≤ 1.0cm (14). Currently, due to advancement in ultrasound technology and the widespread use of FNA, overdiagnosis and overtreatment of thyroid cancer are particularly prevalent (14, 15). Therefore, accurately assessing the nature of thyroid nodules and developing a subsequent diagnosis and treatment plan is crucial. Although ACR-TIRADS is widely used in clinical and can accurately assess the nature of nodules, a single ultrasonic sign still lacks high specificity and sensitivity in diagnosing malignant nodules. The combined application of CEUS and 2017 ACR-TIRADS demonstrated better diagnostic efficiency than their individual application. The CEUS-TIRADS offers a new research direction, namely to analyze the quantitative and qualitative data of CEUS through prospective research and to establish a CEUS classification system for thyroid nodules. In other words, not all malignant nodules exhibit malignant-related ultrasonic signs (16, 17).

The development of CEUS date back to 1969. Gramiak et al. (18). found that an aortic root injection of indocyanine blue could produce an echo enhancement phenomenon in echocardiography. Subsequently, scholars continued to explore to identify ideal contrast agents. Eventually, SonoVue and other contrast agents were widely used in clinical practice. At present, there are significant variations in reports regarding the diagnostic efficacy of CEUS in differentiating benign and malignant thyroid nodules. A meta-analysis of 1,515 thyroid nodules indicated that CEUS has pooled sensitivity, specificity, PPV, and NPV of 85%, 82%, 83%, and 85%, respectively (19). Additionally, different studies have varying interpretations of contrast parameters. Zhang et al. (20) considered that non-uniform enhancement was indicative of malignant lesions, while ring enhancement was more commonly observed in benign nodules. The reason for non-uniform enhancement may be that malignant nodules secrete vascular endothelial growth factor, stimulating the formation of new blood vessels in the nodules and surrounding normal tissues. The fading phase characteristics, including rewind down, retreating, and slow back, have been shown in previous studies to be useful in differentiating benign and malignant thyroid nodules (21, 22). These new vessels often exhibit tortuous and complex patterns, uneven diameters, eccentric distribution and irregular vascular branches. Another reason is that microcalcification in malignant nodules can affect the formation of neovascularization and exacerbate the uneven distribution of blood vessels (20, 23, 24).

In this study, we conducted univariate analysis on the contrast parameters distinguishing benign from malignant nodules. It was observed that non-uniform enhancement, hyper-enhancement, slow entry in enhanced phase, and rewind down in fading phase were typical of malignant nodules. This aligns with previous research findings (25). Logistic regression analysis revealed that non-uniform enhancement was an independent risk factor for predicting malignant nodules, whereas slow entry during the enhancement phase served as an independent protective factor for benign nodules. When CEUS was used alone, the specificity and accuracy for diagnosing malignant thyroid nodules were the lowest, as 50.56% and 75.00%, respectively.

In this study, both the 2017 ACR-TIRADS and CEUS-TIRADS exhibited high diagnostic sensitivity, at 87.27%. The specificity, accuracy, PPV and NPV of CEUS-TIRADS were marginally superior to those of the 2017 ACR-TIRADS and the combined use of CEUS and ACR-TIRADS. In existing studies, there is considerable heterogeneity in reported sensitivity and specificity of 2017 ACR-TIRADS a (26–29). The primary reason for this is the subjectivity among different researchers and the variations in judgment based on their qualification levels.

While our newly proposed CEUS-TIRADS classification demonstrated incremental improvements in specificity (57.30% vs. 55.06%) and Youden index (0.4457 vs. 0.4233) compared with 2017 ACR-TIRADS alone, these differences were not statistically significant (χ² = 0.359, p = 0.949). Therefore, the CEUS-TIRADS should be interpreted as a preliminary scoring approach that offers incremental rather than superior diagnostic value. We innovatively simplified the contrast parameters and assigned scores based on the OR value, providing a framework for future research. This opens up a new research direction—namely, quantifying and characterizing contrast parameters to identify additional indicators in future studies. By establishing a novel CEUS grading system that can be externally validated, we hope to promote the standardization and broader clinical adoption of thyroid CEUS. Recent studies have consistently shown that the integration of CEUS and TIRADS significantly improves the accuracy and specificity of thyroid cancer detection, making this combination a promising diagnostic tool for thyroid cancer (11, 12, 30). Despite our efforts to combine CEUS with TIRADS, the diagnostic efficacy remained suboptimal. Studies have demonstrated that the utilization of multiple ultrasound parameters can enhance thyroid cancer prediction (31). Therefore, in future research, we may consider integrating conventional ultrasound, color Doppler ultrasound, contrast-enhanced ultrasound parameters, and elastography findings to develop a more robust diagnostic predictive model. While the incorporation of CEUS into the TIRADS model resulted in only a minimal increase in specificity and accuracy, we believe that the combination of CEUS and TIRADS offers a more comprehensive approach to thyroid nodule evaluation. Future studies should focus on refining the CEUS-TIRADS model by incorporating additional CEUS parameters and optimizing the scoring system.

### Clinical implications and a risk-stratified algorithm

The diagnostic paradox—intermediate-risk (TIRADS 4) nodules are the most challenging to diagnose yet derive the greatest benefit from CEUS—has direct clinical relevance. Our subgroup analysis confirmed that the incremental value of CEUS was most pronounced in TIRADS 4 nodules (NRI = 0.185), supporting a targeted algorithm in which CEUS is applied selectively to this subgroup rather than universally.

Under this algorithm: TIRADS 1-3 nodules proceed to routine follow-up without CEUS; TIRADS 5 nodules proceed directly to FNA or surgery; and TIRADS 4 nodules undergo CEUS as an intermediate step, with the CEUS-TIRADS score guiding the final FNA decision. This approach offers three advantages: (1) maximizes diagnostic yield in the highest-uncertainty subgroup; (2) improves cost-effectiveness by avoiding CEUS in low- or high-risk patients; and (3) reduces unnecessary FNA biopsies in TIRADS 4 nodules reclassified to lower risk after CEUS. Multicenter prospective validation of this algorithm is warranted before clinical implementation.

### Patient-specific factors influencing CEUS interpretation

Several patient-related factors may affect CEUS interpretation. Hashimoto’s thyroiditis can alter background parenchymal enhancement, potentially confounding the assessment of nodule enhancement intensity. Patient age influences pretest malignancy probability and should be considered when interpreting borderline scores. Nodule location and depth may affect image quality due to attenuation or motion artifacts. These factors should be taken into account in clinical practice, and future studies should incorporate them into personalized diagnostic models to complement the CEUS-TIRADS system.

### Limitations

Several limitations of this study should be acknowledged. First, the study cohort exhibited a substantial imbalance, with 699 malignant nodules (88.7%) and only 89 benign nodules. This predominance of malignant cases reflects the referral bias inherent to our institution as a tertiary referral center, where patients with ultrasonographically suspicious nodules are preferentially referred for CEUS evaluation and subsequent surgical management. This high pretest probability may have inflated the PPV and NPV estimates, while the relatively small number of benign nodules may have reduced the precision of specificity estimates. Consequently, the reported diagnostic performance metrics—particularly PPV (94.13%) and NPV (36.63%)—should be interpreted with caution and may not be generalizable to primary care settings or populations with a lower prevalence of thyroid malignancy. Second, although ring enhancement has been described as a characteristic feature of benign nodules, it was not included as a separate parameter in the current CEUS-TIRADS model. Future studies may consider incorporating ring enhancement into the classification system to further improve specificity. Third, the presence of diffuse thyroid disease, such as Hashimoto’s thyroiditis, may alter the enhancement patterns of the thyroid parenchyma, potentially introducing bias in the interpretation of iso-, hypo-, or hyper-enhancement. We acknowledge that we did not systematically exclude or stratify patients with diffuse thyroid disease in this analysis. Fourth, the single-center retrospective design may limit the generalizability of our findings. Future multicenter prospective studies with a more balanced distribution of benign and malignant nodules are warranted to validate the CEUS-TIRADS scoring system.

## Conclusion

The combined application of CEUS and 2017 ACR-TIRADS demonstrated incremental improvement in diagnostic specificity and Youden index compared with their individual application, though the differences did not reach statistical significance. The CEUS-TIRADS provides a preliminary scoring framework and a promising research direction for future prospective studies to analyze quantitative and qualitative CEUS data and to establish a validated CEUS classification system for thyroid nodules. External validation in larger, more balanced cohorts is warranted before clinical implementation.

### Practical implementation workflow

We propose the following targeted workflow for clinical adoption: perform standard ACR-TIRADS scoring on all nodules; apply CEUS selectively to TIRADS 4 nodules (the subgroup with the greatest diagnostic uncertainty); add 2 points for non-uniform enhancement or fast entry based on OR-derived assignment; calculate the total CEUS-TIRADS score; and use the final category to guide FNA decisions (CEUS-TIRADS 1-3: follow-up; CEUS-TIRADS 4a-4b: consider FNA based on size and patient preference; CEUS-TIRADS 4c-5: recommend FNA or surgery). This algorithm requires no specialized software and is readily implementable in routine practice.

### Future outlook

Several avenues warrant further investigation. Prospective multicenter studies with balanced benign-to-malignant ratios are needed to validate the CEUS-TIRADS system. External validation in populations with lower pretest malignancy probability is essential to assess generalizability. Multi-reader studies should evaluate inter-observer reproducibility across centers and experience levels. Cost-effectiveness analyses comparing CEUS-TIRADS-guided versus conventional TIRADS-guided FNA decisions would inform health-economic value. Future iterations may incorporate additional CEUS features (e.g., ring enhancement, quantitative time-intensity curve parameters) to improve accuracy. Machine learning algorithms could enable automated CEUS feature extraction, reducing operator dependence. Finally, studies examining patient-reported outcomes and long-term follow-up data are needed to determine whether CEUS-TIRADS-guided decisions translate into sustained clinical benefits.

## Data Availability

The original contributions presented in the study are included in the article/supplementary material. Further inquiries can be directed to the corresponding author.
